# High-Sensitive FAM Labeled Aptasensor Based on Fe_3_O_4_/Au/g-C_3_N_4_ for the Detection of Sulfamethazine in Food Matrix

**DOI:** 10.3390/bios12090759

**Published:** 2022-09-15

**Authors:** Xueling Yan, Lulan Yang, Jiaming Tang, Xu Wen, Xingyue Chen, Xiaoling Zheng, Lingling Chen, Jiaqi Li, Tao Le

**Affiliations:** College of Life Sciences, Chongqing Normal University, Chongqing 401331, China

**Keywords:** aptasensor, sulfamethazine, Fe_3_O_4_/Au/g-C_3_N_4_, food matrix

## Abstract

In this study, we developed a fluorescent aptasensor based on Fe_3_O_4_/Au/g-C_3_N_4_ and a FAM-labeled aptamer (FAM-SMZ1S) against sulfamethazine (SMZ) for the specific and sensitive detection of SMZ in food matrix. The FAM-SMZ1S was adsorbed by the Fe_3_O_4_/Au/g-C_3_N_4_ via π–π stacking and electrostatic adsorption, serving as a basis for the ultrasensitive detection of SMZ. Molecular dynamics was used to explain the reasons why SMZ1S and SMZ were combined. This aptasensor presented sensitive recognition performance, with a limit of detection of 0.16 ng/mL and a linear range of 1–100 ng/mL. The recovery rate ranged from 91.6% to 106.8%, and the coefficient of variation (CV) ranged from 2.8% to 13.4%. In addition, we tested the aptasensor for the monitoring of SMZ in various matrix samples, and the results were well-correlated (R^2^ ≥ 0.9153) with those obtained for HPLC detection. According to these results, the aptasensor was sensitive and accurate, representing a potentially useful tool for the detection of SMZ in food matrix.

## 1. Introduction

Sulfamethazine (SMZ) is a member of the sulfonamide family of antibiotics, and it is widely used to treat bacterial infections caused by pathogenic bacteria and parasites in livestock [1]. However, the overuse of SMZ in animal breeding leads to SMZ residues, which then accumulate in the human body through the food chain and cause allergic and toxic reactions [2,3]. The European Union, the United States, and China have established maximum residue limits of 100 μg/kg SMZ [4,5]. In previous studies, researchers have used numerous methods for the detection and quantification of SMZ, including liquid chromatography–mass spectrometry (LC–MS), high-performance liquid chromatography (HPLC), and the enzyme-linked immunosorbent assay (ELISA) [6,7,8]. Although LC–MS and HPLC are sensitive and highly specific for SMZ, they require complicated operations and are not suitable for detection in large numbers of samples [9]. Because ELISA is dependent on the surrounding environment, it may be limited in practice [10]. Therefore, there is an urgent need to construct a rapid and sensitive method for detecting SMZ in food matrix.

Aptamers are 70–100 nt RNA or ssDNA that are generally prepared through systematic evolution of ligands by exponential enrichment (SELEX) and often used as bioprobes, which recognize and bind targets with high affinity and specificity [11]. Aptamers can fold into unique spatial structures and can specifically bind to target molecules, such as cells, proteins, antibiotics, toxins, and other biomarkers, through hydrogen bonds, hydrophobic interactions, and van der Waals forces [12]. Kou screened the shortest aptamer (SMZ1S), which can be used as a capture probe to identify and combine SMZ to detect SMZ in the sample matrix. However, SMZ1S was only used in the matrix of eggs and milk [4], so the performance of SMZ1S also needs to be verified in more sample substrates. Fluorescent aptasensors are widely used in food detection due to their sensitivity, specificity and low cost [13,14]. In our previous research, we constructed an aptasensor -based on Fe_3_O_4_/Au/g-C_3_N_4_ for the sensitive and accurate detection of sulfameter. In this study, because of its excellent fluorescence-quenching ability, we constructed a fluorescent aptasensor based on Fe_3_O_4_/Au/g-C_3_N_4_ to detect SMZ. Previously constructed aptasensors for detecting SMZ are based on graphene oxide (GO) and gold nanoparticles (AuNPs). However, the structure of GO is rich in oxygen-containing groups, which can lead to false-positive detection results for GO-based aptasensors [15,16]. AuNPs are susceptible to salt-induced aggregation, and the adsorption rate of aptamers increases with higher salt concentrations, which makes it difficult to apply AuNPs-based aptasensors to complex biological mechanistic samples [17]. The aptasensor developed in our study bypasses these issues and, moreover, has a lower limit of detection (LOD) and wider ranges in terms of SMZ detection [4,18,19]. However, reports are lacking on the quantitative measurement of SMZ based on the use of Fe_3_O_4_/Au/g-C_3_N_4_.

In this work, we constructed, for the first time, a Fe_3_O_4_/Au/g-C_3_N_4_ fluorescent aptasensor, and the LOD was found to be the lowest value for all aptasensors in SMZ detection. The reason for the high specificity of SMZ1S to SMZ was also investigated by molecular dynamics simulations. We also tested the established aptasensor in the analysis of food matrices, including milk, egg, honey, swine, swine kidney, chicken, chicken liver, beef, crucian, and shrimp, which we spiked with different concentrations of SMZ. Finally, we compared the results of HPLC and this method, and explored their correlation, proving that the aptasensor could detect trace SMZ in multiple complex sample matrix.

## 2. Materials and Methods

### 2.1. Materials

SMZ1S (5′-CGTTAGACG-3′; K_d_ = 24.6 nM) was selected for use as in our previous study [4]. The FAM-labeled SMZ1S (FAM-SMZ1S) was synthesized by Sangon Biotech (Shanghai, China). SMZ and other antibiotic standards as well as the HAuCl_4_·aq and N,N-dimethylformamide (DMF) were purchased from Sigma-Aldrich (St. Louis, MO, USA). Deionized H_2_O (DI H_2_O) was prepared using the Millipore Milli-Q Ultrapure Water System (Bedford, MA, USA).

### 2.2. Synthesis of Fe_3_O_4_/Au/g-C_3_N_4_

**Figure 1:**
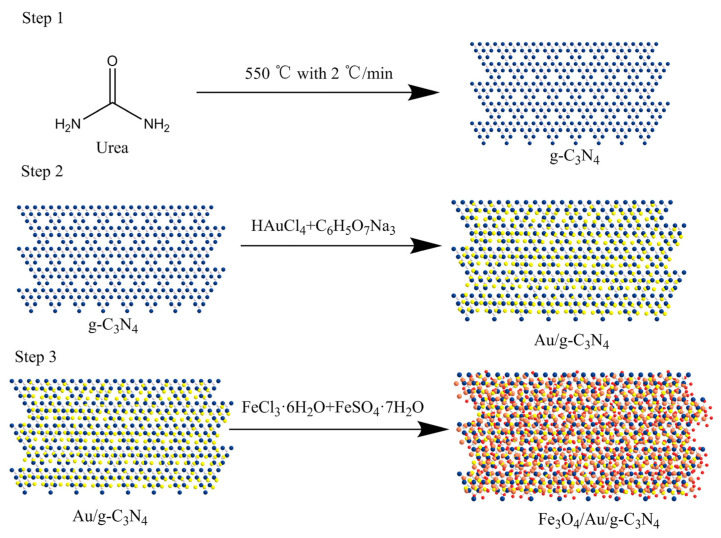
Fe_3_O_4_/Au/g-C_3_N_4_ synthesis path: Step 1: Preparation of g-C_3_N_4_ by heating with urea. Step 2: Preparation of Au/g-C_3_N_4_ by heating g-C_3_N_4_, HAuCl_4_ and C_6_H_5_O_7_Na_3_. Step 3: Preparation of Fe_3_O_4_/Au/g-C_3_N_4_ by heating Au/g-C_3_N_4_, FeCl_3_·6H_2_O and FeSO_4_·7H_2_O.

As shown in Figure 1, Fe_3_O_4_/Au/g-C_3_N_4_ was prepared using a hydrothermal synthesis reaction. Briefly, 15 g urea was gradually heated to 550 °C, with a ramp of 2 °C/min, and the temperature was maintained for 4 h to obtain the yellow solid of g-C_3_N_4_. The g-C_3_N_4_ (1 g) was added to 50 mL of 1.5 mM trisodium citrate solution, and the mixture was sonicated for 20 min. Subsequently, 25 mL of 1 mM chloroauric acid solution was added to the reaction mixture, which was heated at 60 °C for 2 h. Then, the reacted mixture was suction-filtered and washed 4 times with DI H_2_O and alcohol with subsequent freeze-drying to obtain Au/g-C_3_N_4_. Later, 0.15 g Au/g-C_3_N_4_, 0.5 mmol FeCl_3_·6H_2_O, and 0.73 mmol of FeSO_4_·7H_2_O were added to 50 mL DI H_2_O, followed by sonication for 1 h. The mixture was then transferred to a hydrothermal reactor, 3 mmol NaOH was added, and the mixture was then heated at 120 °C for 24 h. After cooling, the mixture was washed 4 times with DI H_2_O followed by ethanol. Finally, we obtained Fe_3_O_4_/Au/g-C_3_N_4_ using magnetic separation and freeze-drying [12,20].

### 2.3. Preparation of the Aaptasensor

To prepare the aptasensor, 1 mL of 1 mg/mL of Fe_3_O_4_/Au/g-C_3_N_4_ was first sonicated for 30 min, followed by adding 10 μL of 1 mg/mL PEG 20,000 and subsequent incubation for 12 h to block nonspecific binding sites, and the resulting mixture was then stored at 4 °C before use. Subsequently, FAM-SMZ1S was diluted to 100 nM with binding buffer (100 mM NaCl, 2 mM MgCl_2_, 20 mM Tris-HCl, 1 mM CaCl_2_, 5 mM KCl, and 0.02% Tween 20, pH 7.6). Then, 199 μL of 100 nM FAM-SMZ1S and 1 μL of different concentrations of SMZ (0, 1, 5, 25, 50, 100 ng/mL) were incubated for 30 min (i.e., to a final volume of 200 μL) at 25 °C in the dark. Along with 60 μL of 1 mg/mL of Fe_3_O_4_/Au/g-C_3_N_4_, the samples were incubated for 5 min. The supernatant was then collected by magnetic separation. We measured the fluorescence-intensity value of the supernatant using Varioskan LUX (Thermo Scientific, Waltham, MA, USA) (λ_ex_ = 492 nm, λ_em_ = 518 nm). The standard curves were constructed based on the fluorescence intensity of SMZ standards at different concentrations of 0, 1, 5, 25, 50, and 100 ng/mL. The LOD was calculated as follows: LOD = 3 SD/slope.

### 2.4. Molecular Dynamics Trajectory Analysis

Equilibrium was achieved under a constant particle number, volume and temperature, as well as optimal isothermal and isobaric conditions [21,22]. The SMZ1S-SMZ complex was then subjected to 60 ns implicit solvent model molecular dynamics (MD) simulations, and the resulting structurally stable complexes were subjected to explicit solvent model molecular dynamics simulations. MD simulations of SMZ1S and other antibiotics, such as sulfamethazine sulfanilamide, sulfameter, sulfadiazine, sulfamethoxypyridazine, chloramphenicol, kanamycin, and chlortetracycline, were performed in the same manner. The root mean square deviation (RMSD) was used as a criterion to evaluate the stability of the aptamer target. All simulation processes were implemented using GROMACS (University of Goettingen, Germany) and G_MMPBSA software [23,24].

### 2.5. Aptasensor Selectivity

To study its selectivity, we tested the performance of the aptasensor in detecting other sulfonamides, such as sulfamethazine, sulfanilamide, sulfameter, sulfadiazine, sulfamethoxypyridazine, and other commonly used antibiotics (i.e., chloramphenicol, kanamycin, and chlortetracycline). For this, 199 μL of 100 nM FAM-SMZ1S was incubated with 1 μL of the different antibiotics at 100 ng/mL for 30 min. Subsequently, 60 μL of 1 mg/mL of Fe_3_O_4_/Au/g-C_3_N_4_ was added to the mixture, which was incubated for 5 min, after which we measured the fluorescence intensity of the supernatant. After measuring the corresponding fluorescence intensity F of different antibiotics and the fluorescence intensity F_0_ of blank sample, and the value of ΔF (ΔF = F − F_0_) was calculated.

### 2.6. Sample Preparation

All samples were purchased from the local supermarket and processed using a previously established procedure with slight modifications [12]. To prepare the milk samples, 2 mL of skimmed milk was centrifuged at 14,000 rpm at 4 °C for 20 min and diluted to 20 mL. The milk was then filtered using a 0.22-μm filter membrane. To prepare the egg sample, 2 g egg and 4 mL ethyl acetate were thoroughly mixed and then centrifuged at 12,000 rpm for 10 min. Subsequently, the ethyl acetate in the mixture was evaporated under a nitrogen flow at 40 °C. To prepare the honey sample, 2 mL of honey was diluted 10 times with PBS (136.89 mM NaCl; 2.67 mM KCl; 8.1 mM Na_2_HPO_4_; 1.76 mM KH_2_PO_4_; pH: 7.4). The remaining samples (5 g each of crucian, shrimp, swine, swine kidney, chicken, chicken liver and beef) were individually processed using a homogenizer, after which 25 mL of acetonitrile was added to the mixture followed by agitation for an additional 15 min. The resulting mixture was sonicated for 10 min and finally centrifuged at 12,000 rpm for 15 min. The supernatant was collected and transferred to 30 mL acetonitrile-saturated n-hexane, followed by agitation for 10 min to remove the fat. Finally, the organic solvents were evaporated by heating the mixture in a water bath at 80 °C, and the dried residue was dissolved in 50 mL of binding buffer and diluted 10 times for use [25,26,27].

### 2.7. Validation of Aptasensor

We validated the performance of the aptasensor by applying it in the detection of 10 food matrices. HPLC was first used to confirm that all the samples were free of SMZ. The standard curves for the food matrices samples were constructed as described in the preparation of the aptasensor above. The accuracy and precision of the aptasensor were evaluated by analyzing the above samples spiked with SMZ at three different levels (50, 100, 200 μg/kg), with each concentration level tested 5 times. To verify the reliability of the aptasensor in the food matrices, we conducted a comparison of the results for the aptasensor and HPLC using the same 10 food matrices. All spiked samples with different concentrations of SMZ were subjected to HPLC analysis according to a published procedure [28]. Linear regression was used to calculate the correlations between the results obtained from both the aptasensor and the HPLC [29].

## 3. Results and Discussion

### 3.1. Fluorescence-Quenching Effect between SMZ1S and Fe_3_O_4_/Au/g-C_3_N_4_

**Figure 2:**
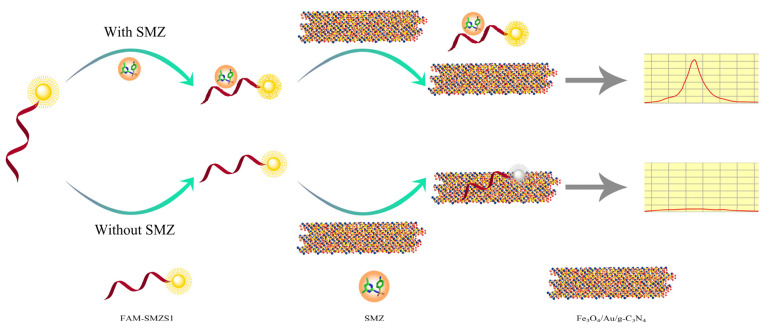
The principle of the aptasensor for SMZ detection.

As shown in Figure 2, in the absence of SMZ, the FAM-SMZ1S was adsorbed by Fe_3_O_4_/Au/g-C_3_N_4_, and the fluorescence was quenched by electron-induced transfer [30], electrostatic adsorption, and π–π stacking. However, in the presence of SMZ, FAM-SMZ1S specifically bound SMZ and would thus not be adsorbed by Fe_3_O_4_/Au/g-C_3_N_4_, which prevented quenching of the fluorescence from FAM-SMZ1S. Therefore, the higher the SMZ concentration, the higher the fluorescence value in the supernatant after magnetic separation. Conversely, when the SMZ concentration decreased, the detected fluorescence value decreased. Thus, the basis for our constructed fluorescence aptasensor for SMZ detection was the functional relationship between the SMZ concentration and the fluorescence value.

### 3.2. Optimization of Detection Conditions

**Figure 3:**
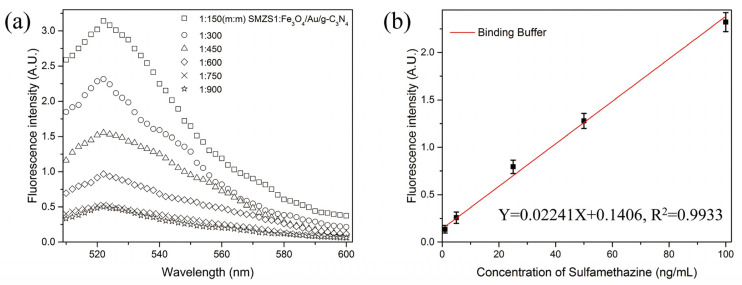
(**a**) Fluorescence emission spectra of SMZ1S:Fe_3_O_4_/Au/g-C_3_N_4_ of different qualities. (**b**) The standard curve of the aptasensor used to detect different concentrations of SMZ in binding buffer.

We constructed the aptasensor by determining the optimized quality ratio between the FAM-SMZ1S and Fe_3_O_4_/Au/g-C_3_N_4_. As shown in Figure 3a, the fluorescence value decreased as the mass ratio of FAM-SMZ1S vs. Fe_3_O_4_/Au/g-C_3_N_4_ decreased. When the mass ratio was reduced to 1:750, the fluorescence value was reduced to a low level, and when the ratio continued to decrease, the fluorescence value did not substantially change. Therefore, we selected the mass ratio of FAM-SMZ1S vs. Fe_3_O_4_/Au/g-C_3_N_4_ at 1:750 as the optimal mass ratio for constructing the fluorescence aptasensor. As shown in Figure 3b, the LOD of the aptasensor was 0.16 ng/mL, and the aptasensor maintained a good linear relationship between 1 and 100 ng/mL (Y = 0.02241 X + 0.1406, R^2^ = 0.9933).

### 3.3. Stability Analysis of Aptamer Target and Selectivity Analysis

**Figure 4:**
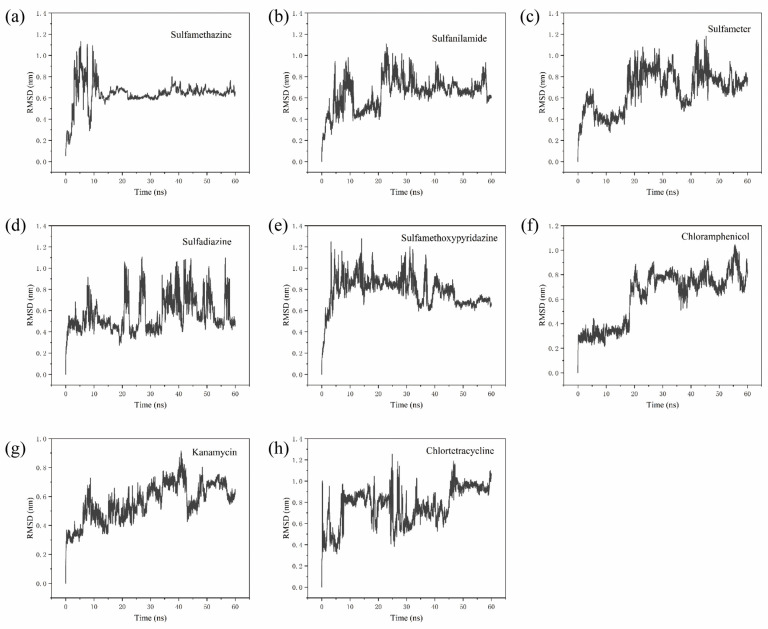
RMSD curve of SMZ1S with SMZ (**a**), sulfanilamide (**b**), sulfameter (**c**), sulfadiazine (**d**), sulfamethoxypyridazine (**e**), chloramphenicol (**f**), kanamycin (**g**), and chlortetracycline (**h**).

**Figure 5:**
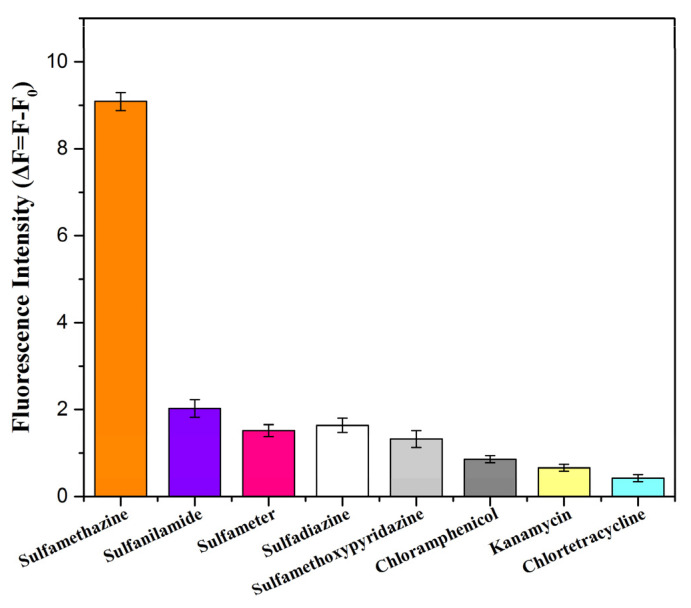
Selectivity of the fluorescent aptasensor for SMZ. Antibiotic standards: sulfamethazine sulfanilamide, sulfameter, sulfadiazine, sulfamethoxypyridazine, chloramphenicol, kanamycin, and chlortetracycline.

After a stable SMZ1S structure was obtained via 60 ns implicit model MD simulations, the RMSD values of the complex SMZ1S system with eight antibiotics, including SMZ, were calculated. As shown in Figure 4, the RMSD value curves of all of the systems fluctuate significantly, besides that of chloramphenicol, showing that there was significant movement of the ssDNA skeleton in the range of 0–15 ns. Furthermore, in the SMZ1S-SMZ system, the overall RMSD value curve fluctuated around 0.6 nm (Figure 4a). As shown in Figure 4e,f, the average of the RMSD values of SMZ1S in sulfamethoxypyridazine and chloramphenicol fluctuated between 0.9 nm and 0.8 nm, but the amplitude was much larger than that of SMZ1S-SMZ. As shown in Figure 4b–h, in the MD simulation of other aptamer target systems, the RMSD values fluctuated greatly. This indicated that the complex formed by SMZ1S and other antibiotics undergoes violent movement during the MD simulation in the range of 0–60 ns, and the complex was unstable. Meanwhile, the selectivity of the aptasensor was validated by the selective testing of different non-targeting antibiotics. As shown in Figure 5, the fluorescence intensity (ΔF) of other sulfonamide antibiotics similar in structure to SMZ was much lower than that of SMZ, while the fluorescence intensity values of other antibiotics were even lower. These results indicate that the aptasensor had great selectivity for SMZ because SMZ1S stably bound to SMZ.

### 3.4. Validation of Aptasensor

**Figure 6:**
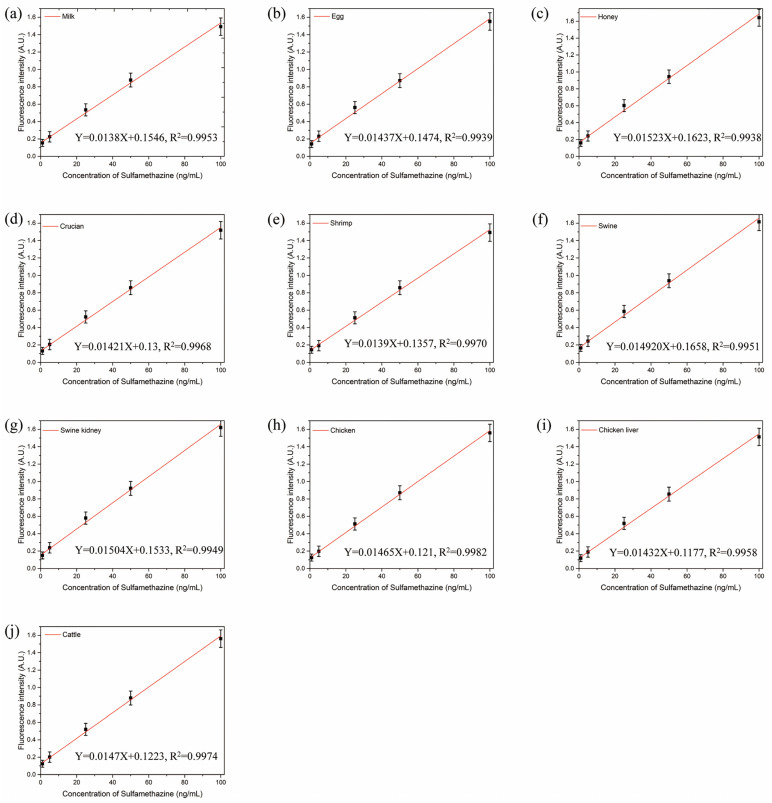
Standard curves corresponding to detection in food matrix, including (**a**) milk, (**b**) egg, (**c**) honey, (**d**) crucian, (**e**) shrimp, (**f**) swine, (**g**) swine kidney, (**h**) chicken, (**i**) chicken liver and (**j**) cattle.

We demonstrated the performances of the aptasensor by detecting the spiked SMZ with different concentrations (i.e., 50, 100, and 200 μg/kg) in food matrices. In Figure 6a–j, we depicted the standard curves in the various matrix samples, including milk, egg, honey, crucian, shrimps, swine, swine kidney, chicken, chicken liver, and cattle. As shown in Table 1, the LODs in the different food matrices ranged from 0.29 to 0.69 ng/mL, the recovery rate of the aptasensor ranged from 91.6% to 106.8%, and the CVs ranged from 2.8% to 13.4%. For HPLC detection, the recovery rate varied from 95.7% to 107.9%, and the CVs varied from 1.5% to 11.9%. The results of the aptasensor positively correlated with those of HPLC (R^2^ ≥ 0.9153). These results demonstrated that the aptasensor can be applied to accurately and sensitively detect SMZ in food matrix.

## 4. Conclusions

We developed, for the first time, a sensitive and accurate fluorescent aptasensor based on FAM-SMZ1S and Fe_3_O_4_/Au/g-C_3_N_4_ to detect SMZ in 10 food matrixes. Through molecular dynamics simulation, in the SMZ1S and SMZ system, the RMSD value fluctuated near 0.6 nm from 15 ns to 60 ns, indicating that SMZ1S had high selective. According to the experimental results, the aptasensor had a satisfactory linear detection range (1–100 ng/mL), a low LOD (0.16 ng/mL), and a high selectivity in SMZ detection. The accuracy of the established aptasensor for the detection of SMZ was specifically demonstrated by the satisfactory correlation (R^2^ ≥ 0.9153) between the aptasensor data obtained in this work and HPLC. Therefore, the aptasensor was a reliable tool and can be used as an analytical means to monitor SMZ in food matrix.

## Figures and Tables

**Table 1:** LODs, Mean recoveries and coefficients of variation in spiked samples and correlations between the aptasensor and HPLC results (*n* = 5).

Sample	LOD (μg/kg)	Spiked (μg/kg)	Fe_3_O_4_/Au/g-C_3_N_4_		HPLC		The Correlations (R^2^)
			Recovery (%)	CV (%)	Recovery (%)	CV (%)	
Milk	0.331	50	97.5	9.5	99.3	5.7	0.9977
		100	97.1	4.2	99.3	5.9	
		200	103.7	3.1	101.1	3.9	
Egg	0.489	50	106.8	10.4	100.9	12.0	0.9756
		100	101.1	5.9	99.0	7.1	
		200	97.7	3.4	98.5	3.7	
Honey	0.294	50	91.6	6.8	104.1	5.2	0.9894
		100	102.7	4.8	95.8	4.6	
		200	100.7	3.9	98.1	4.2	
Crucian	0.518	50	93.4	8.4	103.3	3.9	0.9713
		100	96.9	4.8	99.6	5.3	
		200	97.6	2.8	99.6	4.3	
Shrimp	0.556	50	101.4	12.5	107.9	6.9	0.9663
		100	99.0	3.0	103.9	4.1	
		200	97.4	4.4	98.8	2.6	
Swine	0.469	50	98.2	13.4	99.2	7.9	0.9153
		100	99.2	8.1	101.0	7.8	
		200	98.7	3.7	99.6	3.4	
Swine kidney	0.573	50	94.8	6.6	99.0	6.1	0.9321
		100	94.5	4.2	98.1	4.0	
		200	103.6	3.2	101.1	3.5	
Chicken	0.674	50	93.7	5.9	98.2	10.2	0.9459
		100	101.0	6.0	100.9	7.1	
		200	100.4	6.5	100.0	1.5	
Chicken liver	0.615	50	95.7	9.5	97.6	8.3	0.9586
		100	98.0	4.6	106.3	3.9	
		200	96.6	3.1	99.5	2.2	
Cattle	0.411	50	96.3	3.2	101.9	6.1	0.9995
		100	98.2	4.8	103.9	5.2	
		200	98.4	8.2	104.2	3.5	

SD: standard deviation; CV: coefficient of variation.

## Data Availability

Not applicable.

## References

[1] Wang Y., An Y., Liu Z., Zhou Y., Zhang D. (2020). An exploratory study on the simultaneous screening for residues of chloramphenicol, ciprofloxacin and sulphadimidine using recombinant antibodies. Food Addit. Contam. Part A Chem. Anal. Control Expo. Risk Assess.

[2] Dibbern D., Montanaro A. (2008). Allergies to sulfonamide antibiotics and sulfur-containing drugs. Ann. Allergy Asthma Immunol. Off. Publ. Am. Coll. Allergy Asthma Immunol..

[3] He B., Li M., Li M. (2020). Electrochemical determination of sulfamethazine using a gold electrode modified with multi-walled carbon nanotubes, graphene oxide nanoribbons and branched aptamers. Mikrochim Acta.

[4] Kou Q., Wu P., Sun Q., Li C., Zhang L., Shi H., Wu J., Wang Y., Yan X., Le T. (2021). Selection and truncation of aptamers for ultrasensitive detection of sulfamethazine using a fluorescent biosensor based on graphene oxide. Anal. Bioanal. Chem..

[5] Shi H., Kou Q., Wu P., Sun Q., Wu J., Le T. (2020). Selection and Application of DNA Aptamers Against Sulfaquinoxaline Assisted by Graphene Oxide–Based SELEX. Food Anal. Methods.

[6] Su S., Zhang M., Li B., Zhang H., Dong X. (2008). HPLC determination of sulfamethazine in milk using surface-imprinted silica synthesized with iniferter technique. Talanta.

[7] Peng D., Li Z., Wang Y., Liu Z., Sheng F., Yuan Z. (2017). Enzyme-linked immunoassay based on imprinted microspheres for the detection of sulfamethazine residue. J. Chromatogr. A.

[8] Er Demirhan B., Demirhan B. (2022). Detection of Antibiotic Residues in Blossom Honeys from Different Regions in Turkey by LC-MS/MS Method. Antibiotics.

[9] Gao Z., Du X., Ding Y., Li H. (2021). Establishment of a dual-aptasensor for simultaneous detection of chloramphenicol and kanamycin. Food Addit. Contam. Part A Chem. Anal. Control Expo Risk Assess.

[10] Song K.M., Jeong E., Jeon W., Jo H., Ban C. (2012). A coordination polymer nanobelt (CPNB)-based aptasensor for sulfadimethoxine. Biosens. Bioelectron..

[11] Yu Q., Li M., Liu M., Huang S., Wang G., Wang T., Li P. (2021). Selection and Characterization of ssDNA Aptamers Targeting Largemouth Bass Virus Infected Cells With Antiviral Activities. Front. Microbiol..

[12] Yan X., Wang Y., Kou Q., Sun Q., Tang J., Yang L., Chen X., Xu W., Le T. (2022). A novel aptasensor based on Fe_3_O_4_/Au/g-C3N4 for sensitive detection of sulfameter in food matrices. Sens. Actuators B Chem..

[13] Zheng X., Gao S., Wu J., Hu X. (2021). A Fluorescent Aptasensor Based on Assembled G-Quadruplex and Thioflavin T for the Detection of Biomarker VEGF165. Front. Bioeng. Biotechnol..

[14] Suo Z., Liang X., Jin H., He B., Wei M. (2021). A signal-enhancement fluorescent aptasensor based on the stable dual cross DNA nanostructure for simultaneous detection of OTA and AFB. Anal. Bioanal. Chem..

[15] Jiang Y.J., Wang N., Cheng F., Lin H.R., Zhen S.J., Li Y.F., Li C.M., Huang C.Z. (2020). Dual Energy Transfer-Based DNA/Graphene Oxide Nanocomplex Probe for Highly Robust and Accurate Monitoring of Apoptosis-Related microRNAs. Anal. Chem..

[16] Liu Z., Chen S., Liu B., Wu J., Zhou Y., He L., Ding J., Liu J. (2014). Intracellular detection of ATP using an aptamer beacon covalently linked to graphene oxide resisting nonspecific probe displacement. Anal. Chem..

[17] Liu J. (2012). Adsorption of DNA onto gold nanoparticles and graphene oxide: Surface science and applications. Phys. Chem. Chem. Phys..

[18] Wang Y., Yan X., Kou Q., Sun Q., Wang Y., Wu P., Yang L., Tang J., Le T. (2021). An Ultrasensitive Label-Free Fluorescent Aptasensor Platform for Detection of Sulfamethazine. Int. J. Nanomed..

[19] Yang L., Ni H., Li C., Zhang X., Wen K., Ke Y., Yang H., Shi W., Zhang S., Shen J. (2019). Development of a highly specific chemiluminescence aptasensor for sulfamethazine detection in milk based on in vitro selected aptamers. Sens. Actuators B Chem..

[20] Liu G., Wang S., Gondal M., Shen K., Xu Q. (2019). Enhanced Visible Light Photocatalytic Performance of G-C_3_N_4_ Photocatalysts Co-Doped with Gold and Sulfur for Degradation of Persistent Pollutant (Rhodamine B). J. Nanosci. Nanotechnol..

[21] Tang J., Kou Q., Chen X., Wang Y., Yang L., Wen X., Zheng X., Yan X., Le T. (2022). A novel fluorescent aptasensor based on mesoporous silica nanoparticles for the selective detection of sulfadiazine in edible tissue. Arab. J. Chem..

[22] Kumari R., Kumar R., Lynn A., Open Source Drug Discovery Consortium (2014). g_mmpbsa—A GROMACS tool for high-throughput MM-PBSA calculations. J. Chem. Inf. Model..

[23] Chen X., Yang L., Tang J., Wen X., Zheng X., Chen L., Li J., Xie Y., Le T. (2022). An AuNPs-Based Fluorescent Sensor with Truncated Aptamer for Detection of Sulfaquinoxaline in Water. Biosensors.

[24] Autiero I., Ruvo M., Improta R., Vitagliano L. (2018). The intrinsic flexibility of the aptamer targeting the ribosomal protein S8 is a key factor for the molecular recognition. Biochim. Biophys. Acta Gen. Subj..

[25] Wang Z., Hu S., Bao H., Xing K., Liu J., Xia J., Lai W., Peng J. (2021). Immunochromatographic assay based on time-resolved fluorescent nanobeads for the rapid detection of sulfamethazine in egg, honey, and pork. J. Sci. Food Agric..

[26] Sun Y., Dai Y., Zhu X., Han R., Wang X., Luo C. (2019). A nanocomposite prepared from bifunctionalized ionic liquid, chitosan, graphene oxide and magnetic nanoparticles for aptamer-based assay of tetracycline by chemiluminescence. Mikrochim. Acta.

[27] Preetham E., Lakshmi S., Wongpanya R., Vaseeharan B., Arockiaraj J., Olsen R.E. (2020). Antibiofilm and immunological properties of lectin purified from shrimp Penaeus semisulcatus. Fish Shellfish. Immunol..

[28] Zhang X., He K., Fang Y., Cao T., Paudyal N., Zhang X.F., Song H.H., Li X.L., Fang W.H. (2018). Dual flow immunochromatographic assay for rapid and simultaneous quantitative detection of ochratoxin A and zearalenone in corn, wheat, and feed samples. J. Zhejiang Univ. Sci. B.

[29] Zhang J., Li W., Zhu W., Yang Y., Qin P., Zhou Q., Lu M., Cai Z. (2019). Mesoporous graphitic carbon nitride as an efficient sorbent for extraction of sulfonamides prior to HPLC analysis. Mikrochim. Acta.

[30] Wang Q., Wang W., Lei J., Xu N., Gao F., Ju H. (2013). Fluorescence quenching of carbon nitride nanosheet through its interaction with DNA for versatile fluorescence sensing. Anal. Chem..

